# Epigenetic activation of hepatocyte growth factor is associated with epithelial-mesenchymal transition and clinical outcome in non-small cell lung cancer

**DOI:** 10.7150/jca.30034

**Published:** 2019-08-28

**Authors:** Jun Yin, Weimin Hu, Xingyang Xue, Wenfan Fu, Lu Dai, Zeyong Jiang, Shengpeng Zhong, Boyun Deng, Jian Zhao

**Affiliations:** 1 Department of Chest Surgery, Affiliated Cancer Hospital & Institute of Guangzhou Medical University, Guangzhou, Guangdong, China; 2 Department of Abdominal Surgery, Affiliated Cancer Hospital & Institute of Guangzhou Medical University, Guangzhou, Guangdong, China

**Keywords:** non-small cell lung cancer, HGF, DNA methylation, metastasis, biomarker

## Abstract

Hepatocyte growth factor (HGF) expression is repressed in normal differentiated lung epithelial cells, but its expression is aberrantly upregulated in non-small cell lung cancer (NSCLC) and acts as a poor prognostic factor. The underlying molecular mechanisms of aberrant HGF expression are unclear. In this study, a novel differential methylation region located in the HGF promoter was identified, which was associated with aberrant HGF expression in NSCLC. The correlations of HGF promoter methylation detected by methylation specific PCR and HGF expression detected by immunohistochemistry with clinical outcomes were assessed in NSCLC patients. DNA methylation of the HGF promoter was correlated with the activation of HGF expression, which induced epithelial-mesenchymal transition, cell migration and invasion. According to the clinical correlation analysis in 63 NSCLC patients, those with high methylation were more likely to have stages III and IV (51.6% vs. 25.0%, *P*<0.05) and metastasis (57.5% vs. 16.7%, *P*<0.05) than patients with low methylation. In addition, compared with the protein marker of HGF expression, the DNA methylation marker of the HGF promoter had higher specificity for prognostic analysis of metastases in NSCLC. Our study indicated the regulatory mechanisms related to DNA methylation of the HGF promoter for HGF expression in NSCLC epithelial cells, and suggested that the DNA methylation signature of the HGF promoter could potentially be employed as a biomarker to improve the prognostic accuracy of NSCLC.

## Introduction

Lung cancer has the highest morbidity and mortality in the world, and approximately 80% of cases have non-small cell lung cancer (NSCLC). In NSCLC, over 90% of treatment failure and mortality is due to metastasis which refers to the malignant tumor cells leave the primary site and form tumors in lymph nodes and distant organs through lymphatic vessels, blood vessels or body cavity. Normally, the primary tumor is unlikely to kill, but metastasis will result in mortality 1-3. Therefore, research on metastases is important for the successful treatment of NSCLC, although this research is still in the exploratory stage 4, 5.

As reported in recent studies, metastasis is a multistep process 6, 7. In NSCLC, the initial and most critical steps of metastasis include tumor epithelial cells gaining the ability to detach from the primary tumor and enter surrounding tissue or lymphatic/blood vessels. Therefore, as the tumor epithelial cells of NSCLC must be transformed into a mesenchymal phenotype during the metastasis process, the occurrence of epithelial-mesenchymal transition (EMT) is essential for NSCLC metastasis 8-11. Recently, aberrant high expression of hepatocyte growth factor (HGF) was found to be closely correlated with EMT and metastasis in various cancers including NSCLC 12-14. The biological effect of HGF is mediated by specific binding to its transmembrane receptor MET, and then, HGF activates MET carboxyl terminal tyrosine phosphorylation and thus activates cells within a variety of downstream signaling pathways, including adhesion, motility, growth, survival and morphogenesis 15-17. Normally, the expression of HGF is cell-specific, and it was found that HGF is not expressed in the epithelial cells of normal lung tissues 18. However, the underlying basis of aberrant HGF expression in the epithelial cells of NSCLC tissues remains elusive.

In addition to genetic alterations, epigenetic alteration has recently been acknowledged as an equally important mechanism in carcinogenesis 19. In general, epigenetic modifications include DNA methylation, histone modifications, nucleosome positioning, and the regulation of non-coding RNAs. Abnormalities in any of these four epigenetic modifications affect chromatin organization and gene expression, and are also implicated in diseases including cancer 20-22. DNA methylation is a usual covalent modification of cytosines in CpG dinucleotides and can regulate the structure and activity of chromatin 23. Recently, it was found that aberrant DNA methylation in the gene promoter can result in aberrant gene expression in various cancers including NSCLC 24-26. Unlike DNA sequence changes, many epigenetic changes are reversible, and this provides optimism for the treatment of disease through epigenetic modification 27, 28.

As DNA methylation is known to exert an influence on the transcriptional properties of genes in mammals, we assessed the DNA methylation profiles of NSCLC samples and identified a novel differential methylation region in the HGF promoter. HGF promoter, which can control the transcriptional expression of HGF, usually include 1000bp upstream of the transcriptional starting site (TSS) and 5' untranslated region (5'UTR). Interestingly, DNA methylation of the HGF promoter was involved in the activation of HGF expression, which induced EMT, cell migration and invasion in NSCLC epithelial cells. Moreover, compared with immunohistochemistry (IHC) detection of HGF expression in primary NSCLC tissues, the methylation-specific PCR (MSP) detection of HGF promoter methylation levels significantly correlated with advanced tumor stage and metastasis. Thus, these results not only shed light on understanding the mechanisms of aberrant high HGF expression in NSCLC epithelial cells, but have also led to the discovery of a biomarker that can be used to identify NSCLC patients with a higher risk of metastasis.

## Materials and Methods

### Cell lines and cell culture

Human non-small cell lung cancer cells (NSCLC cells) were cultured in RPMI 1640 medium supplemented with 10% fetal bovine serum at 37°C in a humidified atmosphere with 5% CO_2_. A549, HCC827, H460 and H1975 cells were purchased from the company. 16HBE and BEAS-2B cells were given from other laboratory. A549/DDP cells (cisplatin-resistant A549 cells) were induced using progressive concentrations of cisplatin as previously described 29. When the induced cells survived in 6 mmol/L of cisplatin for approximately 2 months with normal activity, the cells were confirmed to be cisplatin-resistant and named A549/DDP cells.

### Tissue sample collection

Primary NSCLC tissues were collected from the Cancer Center of Guangzhou Medical University (Guangzhou, China) with informed consent and Institutional Review Board (IRB) permission. 68 NSCLC patients were recruited into this study, 5 were used only for the methylation array and 63 for validation. All of the following criteria were met: patients who suffered from primary NSCLC; a histological diagnosis of NSCLC with at least one measurable lesion; a TNM clinical stage of I to IV. Patient characteristics are shown in Table S4. Fresh NSCLC tissues were obtained at surgery or by aspiration biopsy and immediately snap-frozen in liquid nitrogen and stored at -80°C until use. All clinical and biological data on these samples were available.

All patients provided written informed consent, and the collection of NSCLC tissues for research purposes was approved by the relevant human research ethics committees of the Cancer Center of Guangzhou Medical University (Approval no. (2014) 100).

### DNA Methylation Assays

According to the manufacturer's instructions, total DNA in cells and tissue samples were extracted using the Genomic DNA Purification Kit (Promega, USA) and were then bisulfite-modified using the EpiTect Bisulfite kit (Qiagen, Germany).

Genome-wide methylation analysis was performed using the validated Illumina Infinium HumanMethylation 450k BeadChip. The methylation score of each CpG is represented as a *β*-value (*β*-value = Max(Signal B, 0)/(Max(Signal A, 0) + Max(Signal B, 0) + 100)).

A pyrosequencing assay was designed to detect the HGF promoter at positions 81770207-81770363 (2 CpGs in the TSS200 region) and 81770004-81770118 (3 CpGs in the 5'UTR region) (chromosome 7, GRCh38/hg38). The sequence reaction and detection were performed by pyrosequencing following the manufacturer's protocol. The results are reported as percentage of the methylated (C) allele over the background of un-methylated (T) allele (Methylation level = mC/(mC + umC) * 100%). The primers for HGF were as follows:

2 CpGs in the TSS200 region: PCR Forward (5' biotinylation) 5'-GATAGGAGTTATTGGGATTTGGAGTTTTAG-3'; PCR Reverse 5'-CCCTTCAACAACACCAAACAAAT-3'; Sequencing 5'-ACAACCCCCCCCATT-3'.

3 CpGs in the 5'UTR region: PCR Forward 5'-AGGAGATGTTTGGGTGAAAG-3'; PCR Reverse (5' biotinylation) 5'-CTTTCCAATTAATCACACAACAAACTTA-3'; Sequencing 5'-TGTTTGGGTGAAAGAAT-3'.

Methylation-specific PCR (MSP) and unmethylation-specific PCR (UMSP) was used to specifically amplify either the methylated or unmethylated HGF promoter after bisulfite conversion. Template negative samples and samples containing genomic DNA not subjected to bisulfite conversion were used as negative controls for both MSP and UMSP. A methylation index (MI) was calculated as follows: MI = [(methylated peak intensity)/(methylated peak intensity + unmethylated peak intensity)].

Primer sequences for MSP and USP were as follows:

MSP primer: Forward 5'-CGTAATAAAAAGTAGTTTAGAGTCGA-3'; Reverse 5'-CATAATACTACTAAACGAACTAACGAA-3'.

UMSP primer: Forward 5'-TGTAATAAAAAGTAGTTTAGAGTTGA-3'; Reverse 5'-CACATAATACTACTAAACAAACTAACAAA-3'.

### Immunohistochemistry (IHC)

5 mm tissue microarray slides were deparaffinized with xylene and ethanol, and antigen retrieval was performed using citrate buffer (pH 6.0) pressure-cooking. Rabbit polyclonal anti-HGF primary antibody (Cell Signaling USA, 1:1000 dilution) was added and incubated overnight at 4°C, and then, streptavidin-horseradish peroxidase conjugate goat-anti-rabbit secondary antibody was added and incubated for 4 h. Staining was visualized using diaminobenzidine and counterstained with acidified hematoxylin.

Histospots with <10% of their area covered by tumor were excluded from analysis. Scoring was performed independently in a blinded manner by two independent observers, and histocores with discrepant scores were re-examined by both observers to achieve a consensus score. Immunostaining was scored on a scale of 0 to 3+ using the following scoring criteria: negative (0), absent staining; weak staining (1+), stronger intensity in < 10% of cancer cells; moderate staining (2+), stronger intensity in 10% to 90% of cancer cells; intense staining (3+), stronger intensity in more than 90% of cancer cells.

### Real-time quantitative PCR (RT-PCR)

Total RNA in cells was extracted using Trizol (Invitrogen, USA). 1 µg total RNA was used for cDNA synthesis using a Reverse Transcription Kit (Takara, Japan), the cDNA was then used for RT-PCR using the SYBR Green Real-time PCR Master Mix (Toyobo, Japan). RT-PCR was performed using the ABI ViiATM7Dx Real-Time PCR System (Life Technologies, USA). Relative expression of mRNA was normalized by β-Actin.

The mRNA RT-PCR primers were designed as follows:

HGF: Forward 5'-ACAGCTTTTTGCCTTCGAGC-3'; Reverse 5'-GCAAGAATTTGTGCCGGTGT-3'.

E-cadherin: Forward 5'-TCATGAGTGTCCCCCGGTAT-3'; Reverse 5'-TCTTGAAGCGATTGCCCCAT-3'.

Vimentin: Forward 5'-GGACCAGCTAACCAACGACA-3'; Reverse 5'-AAGGTCAAGACGTGCCAGAG-3'.

β-Actin: Forward 5'-AGCGAGCATCCCCCAAAGTT-3'; Reverse 5'-GGGCACGAAGGCTCATCATT-3'.

### Western blotting

The cells were harvested and lysed by RIPA buffer for 30 min at 4°C. 50 µg proteins were loaded into 15% SDS-PAGE for analysis. Rabbit polyclonal anti-HGF, anti-E-cadherin, anti-Vimentin or anti-β-Actin primary antibody (Cell Signaling USA, 1:1000 dilution) was added and the cells were incubated overnight at 4°C. Then, HRP (horseradish peroxidase) conjugate goat-anti-rabbit secondary antibody (Cell Signaling USA, 1:1000 dilution) was added and incubated for 4 h. The bound antibodies were detected using the ECL Plus Western Blotting Detection system (GE Healthcare). β-Actin was used as an internal control.

### Transwell assay

The invasion capability of cells was detected using transwell-chamber culture systems (Becton Dickinson, USA). After 48 h incubation at 37°C, the cells were transferred into the upper chamber of the transwell with matrigel (1×10^5^ cells per well in an 8 μm 24-well transwell). Following 24 h incubation at 37°C, cells on the upper surface of the upper chamber (non-invasion cells) were removed by cotton swabs, and cells on the lower surface of the filters were fixed and stained with Giemsa stain. The number of invaded cells was counted under a light microscope (Leica, Germany).

### Scratch assay (wound healing assay)

The migratory ability of cells was assessed by the scratch assay. Cells were grown to confluence in medium containing 10% FBS. A uniform scratch defect was created across the monolayer using a pipette tip. Wells were then washed with PBS, followed by the addition of serum-free medium. Plates were imaged at 0 h, 12 h and 24 h, and the degree the cells at the scratch margin had migrated close to the initial defect was assessed.

### 5-Aza-2'-deoxycytidine (5-Aza-dC) treatment

The DNA methyltransferase inhibitor 5-aza-2'-deoxycytidine (5-Aza-dC) (Sigma) was used to block DNA methylation. Cells were treated with 5-Aza-dC at 10 μM for 48 h. The drug and culture medium were refreshed every day during treatment.

### Chromatin immunoprecipitation (ChIP)

For ChIP analysis, cells grown on a 6-well plate were processed as described in the ChIP Assay kit protocol (Millipore). Chromatin DNA was extracted and broken into fragments of 200-400 bp in length by sonication. The chromatin fragments were then immunoprecipitated with the following antibodies: IgG and anti-RNA polymerase II (anti-RNA Pol II) (Abcam). The precipitated DNA fragments were measured by RT-PCR. To normalize PCR efficiency, the intensity of the PCR products from the chromatin immunoprecipitates were normalized against the intensity of the PCR products of the genomic DNA input amplified by the same primer pairs.

Primers specific for the HGF promoter region (-157 to +13 bp) were as follows:

F/R: Forward 5'-TTTGTAAGTTTCTTTCCTAAGCGT-3'; Reverse 5'-GGTCTGAACTCCCTCTTACGG-3'.

### Statistical analysis

All values were expressed as mean ± standard deviation (SD) from at least three separate experiments. The Student's unpaired t-test, Mann-Whitney *U* test, chi-square test, log-rank statistic, Spearman's correlation, receiver operating characteristic analysis and Kaplan-Meier survival analysis were performed using SPSS 21.0 statistical software (IBM). A two-tailed *P* value test was used in all analyses, and the difference was considered statistically significant if the *P* value was less than 0.05 (*P*<0.05).

## Results

### DNA methylation of the HGF promoter in NSCLC

**Figure 1:**
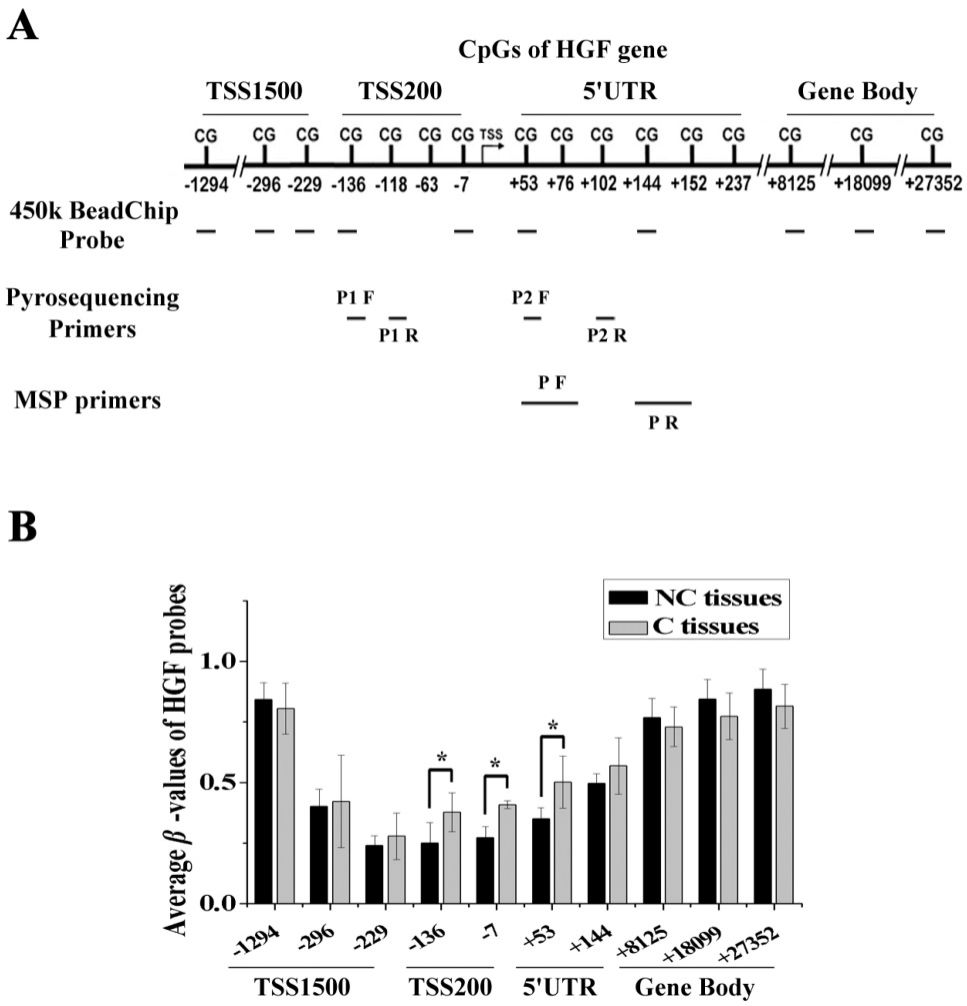
Identification of a novel differential methylation region around the HGF promoter in NSCLC. **(A)** The location of Illumina Infinium HumanMethylation 450k BeadChip HGF probes. **(B)** Average *β*-values of probes mapping to different regions of the HGF promoter in C and NC tissues. C, carcinoma tissues; NC, paracarcinoma tissues. (n=3, *P < 0.05)

We performed DNA methylation profiling in 5 pairs of NSCLC samples (carcinoma tissues, C, and paracarcinoma tissues, NC) using the Illumina Infinium 450k BeadChip, and there were 10 probes mapped to different regions associated with the HGF gene. These regions were defined as 1500bp upstream of the transcription start site (TSS1500), 200bp upstream of the TSS (TSS200), 5' untranslated region (5'UTR), and gene body (Fig. 1A). As the gene expression state was influenced by different gene regions, we compared the *β*-values of probes mapping to different regions between C and NC tissues. The results showed that several of the top differential probes (fold change > 1.5 or < 0.67) were mapped to the TSS200 region, followed by the 5'UTR region, which are around the promoter region of HGF gene (Fig. 1B and Table S1). Therefore, DNA methylation profiling analysis revealed the presence of a novel differential methylation region around the HGF promoter in NSCLC, which indicated that increased DNA methylation of the HGF promoter may occur during the development of NSCLC.

### DNA methylation of the HGF promoter is associated with the activation of HGF expression in NSCLC epithelial cells

**Figure 2:**
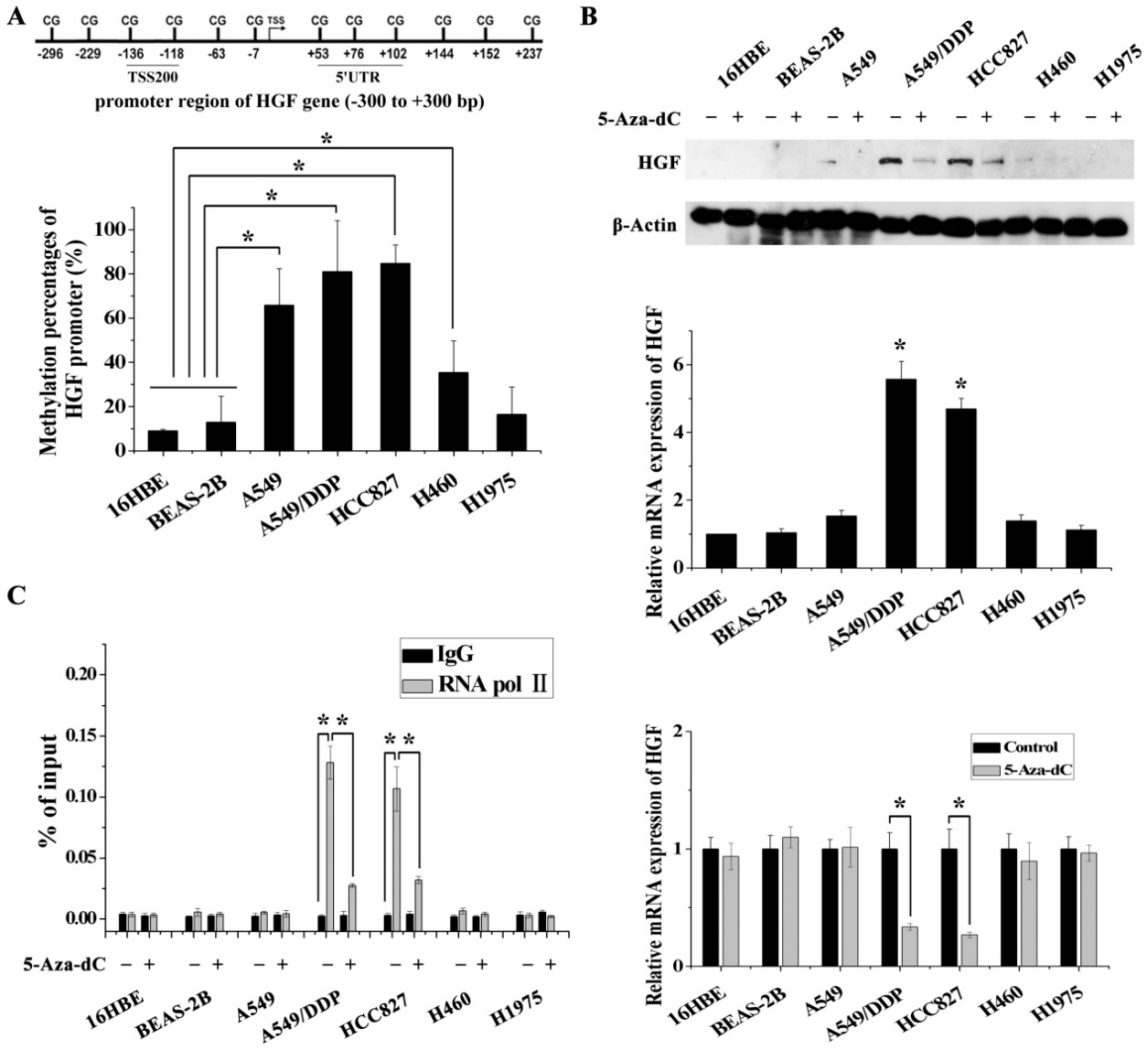
DNA methylation of the HGF promoter region was associated with activation of HGF expression. **(A)** Statistical analysis of the percentage of methylated CpGs in the HGF promoter. **(B)** mRNA and protein expression levels of HGF in 5 NSCLC epithelial cell lines (A549, A549/DDP, HCC827, H460 and H1975) and 2 normal lung epithelial cell lines (16HBE and BEAS-2B), and analysis of HGF mRNA and protein expression levels following treatment with the demethylating agent, 5-aza-2'-deoxycytidine (5-Aza-dC), in these cells. **(D)** RNA polymerase II (RNA Pol II) signal in the HGF promoter was detected by chromatin immunoprecipitation (ChIP) assay, and analysis of the effect of 5-Aza-dC treatment in these cells. (n=3, *P < 0.05)

In order to validate the impact of HGF promoter methylation on HGF expression, we determined promoter methylation status, mRNA and protein expression in 5 NSCLC epithelial cell lines (A549, A549/DDP, HCC827, H460 and H1975) and in 2 normal lung epithelial cell lines (16HBE and BEAS-2B). Firstly, we performed pyrosequencing analysis to detect the methylation status of 2 CpGs in the TSS200 region and 3 CpGs in the 5'UTR region of the HGF gene (Fig. 1A and 2A). The results showed that the average percentage methylation of 5 CpGs around the HGF promoter was high (>80%) in A549/DDP and HCC827 cells, medium (20%-80%) in A549 and H460 cells, and low (<20%) in H1975, 16HBE, and BEAS-2B cells (Fig. 2A, Fig. S1 and Table S2). Using Western blotting and RT-PCR, HGF expression level was found to be high in A549/DDP and HCC827 cells (high methylation), and low in A549, H460, H1975, 16HBE, and BEAS-2B cells (medium or low methylation) (Fig. 2B). To further confirm the effect of DNA methylation on HGF expression, the inhibitor of DNA methyltransferase, 5-Aza-dC, was used to reduce the methylation levels of the HGF promoter, and the HGF expression was found to be downregulated in A549/DDP and HCC827 cells (Fig. 2C). Moreover, it is well known that RNA polymerase II (RNA Pol II) with an affinity for the gene promoter can effect transcriptional activation. Therefore, we speculated that the binding of RNA Pol II to the HGF promoter may be influenced by DNA methylation status. To test this hypothesis, we performed chromatin immunoprecipitation (ChIP) assays using RNA Pol II antibody and control IgG in the different NSCLC cell lines. The results showed that RNA Pol II specifically and strongly bound to the HGF promoter in A549/DDP and HCC827 cells (high methylation) compared with A549, H460, H1975, 16HBE, and BEAS-2B cells (medium or low methylation) (Fig. 2D). Moreover, 5-Aza-dC treatment also resulted in decreased binding of RNA Pol II in A549/DDP and HCC827 cells (Fig. 2D). Based on these results, normal lung epithelial cells have low HGF promoter methylation and low HGF expression, however, some NSCLC epithelial cells can have high HGF promoter methylation and high HGF expression, indicating that DNA methylation may be one of the regulatory mechanisms contributing to the activation of HGF expression in NSCLC epithelial cells.

### Activation of HGF expression induced by DNA methylation of the HGF promoter is involved in regulating EMT in NSCLC epithelial cells

**Figure 3:**
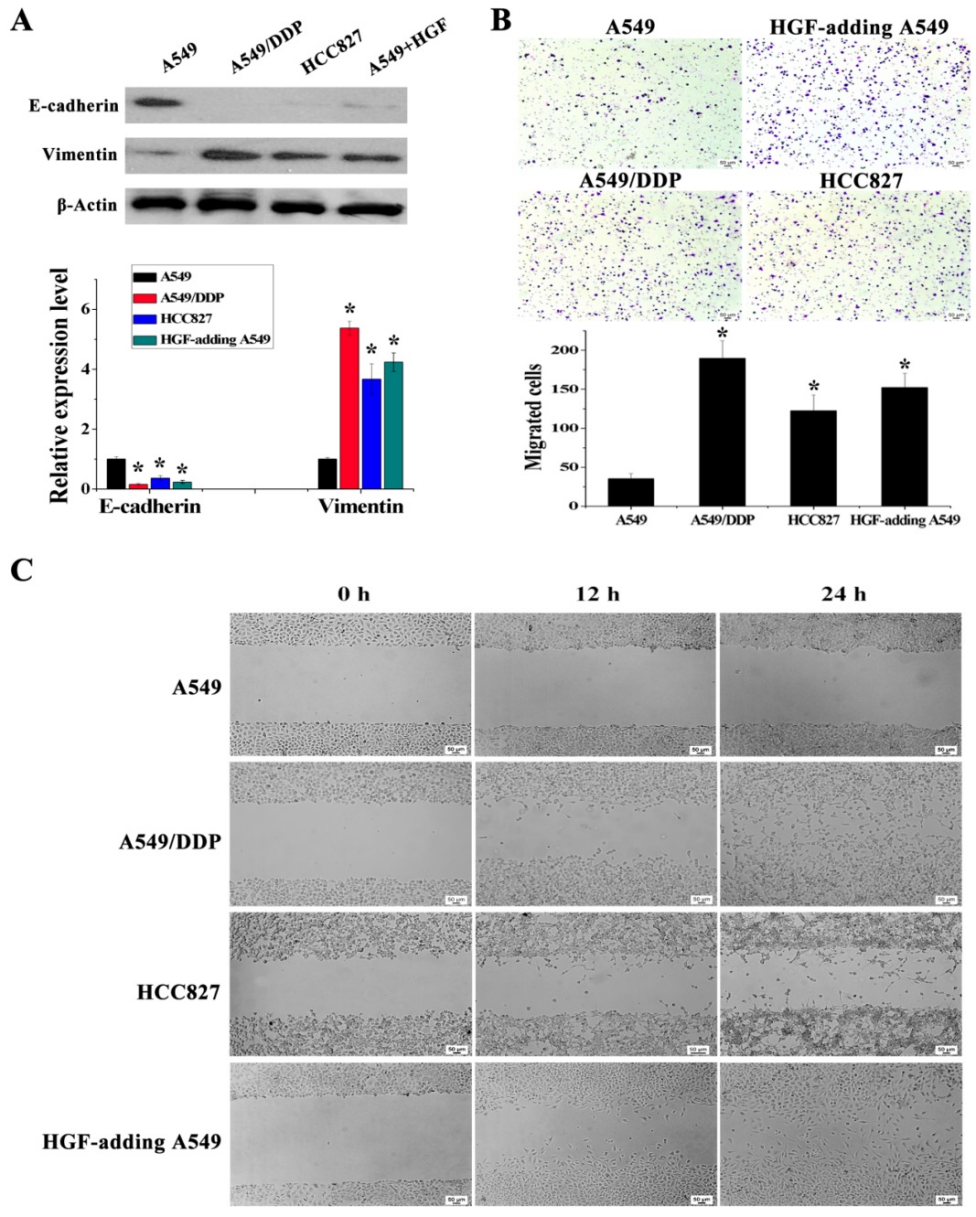
HGF activated by HGF promoter methylation can induce EMT, cell invasion and migration. **(A)** mRNA and protein expression levels of E-cadherin and Vimentin in A549, A549/DDP, HCC827 cells and HGF-adding A549 cells were measured by RT-PCR and Western blotting. **(B)** Cell invasion in these cells was detected by transwell-chamber culture systems. Bar graphs show the number of invaded cells. **(C)** Cell migration in these cells was detected by the scratch assay.

**Figure 4:**
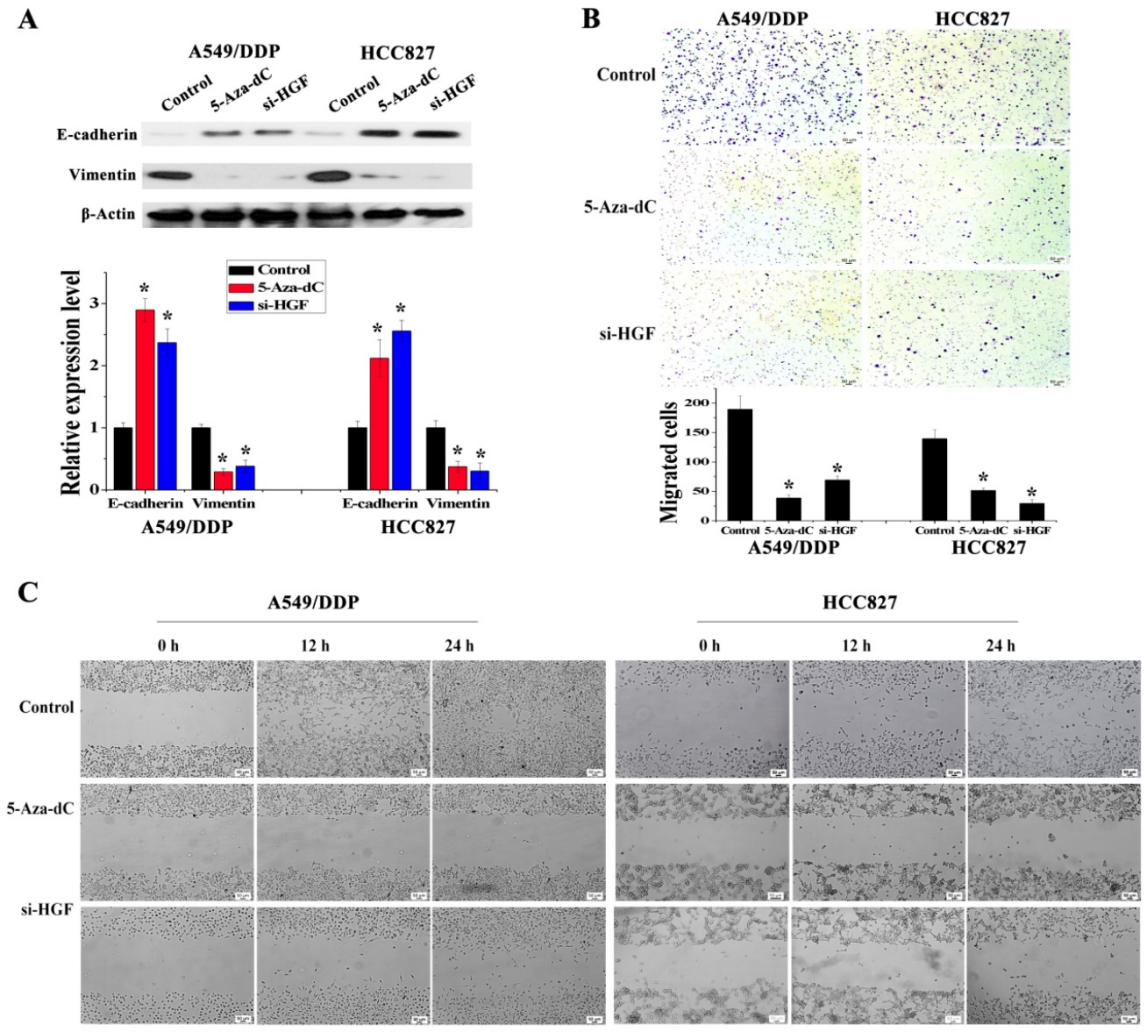
Analysis of the effect of 5-Aza-dC treatment on HGF induced EMT, cell invasion and migration.** (D)** mRNA and protein expression levels of E-cadherin and Vimentin in A549/DDP and HCC827 cells with or without 5-Aza-dC or si-HGF treatment. **(E)** Cell invasion in A549/DDP and HCC827 cells with or without 5-Aza-dC or si-HGF treatment. **(F)** Cell migration in A549/DDP and HCC827 cells with or without 5-Aza-dC or si-HGF treatment. (n=3, *P < 0.05)

It has been reported that overexpression of HGF can induce EMT and promote the migration and invasion of tumor epithelial cells, including NSCLC epithelial cells 13, 14. As mentioned above, some NSCLC epithelial cells with high HGF promoter methylation such as A549/DDP and HCC827 cells can activate the expression of HGF. Moreover, A549/DDP cells were derived from A549 cells which have medium HGF promoter methylation and low HGF expression. Therefore, A549/DDP, HCC827 and A549 cells were used to assess whether the activation of HGF expression induced by DNA methylation of the HGF promoter was involved in the regulation of EMT, cell migration and invasion in NSCLC epithelial cells. Compared with A549 cells, the epithelial marker E-cadherin was decreased and the mesenchymal marker Vimentin was increased in A549/DDP and HCC827 cells, and exogenous addition of HGF to A549 cells (HGF-adding A549) also resulted in a decrease in E-cadherin and an increase in Vimentin (Fig. 3A). Moreover, according to the results of the cell migration and invasion assays, the migration and invasion ability of A549/DDP, HCC827 cells and HGF-adding A549 cells was also significantly increased compared with A549 cells (Fig. 3B and 3C). Furthermore, we also used 5-Aza-dC to inhibit HGF promoter methylation and introduced siRNA targeting HGF (si-HGF) to inhibit HGF expression in A549/DDP and HCC827 cells. Following treatment of A549/DDP and HCC827 cells with 5-Aza-dC and si-HGF, E-cadherin was increased and Vimentin was decreased (Fig. 4A). In addition, the migration and invasion ability of A549/DDP and HCC827 cells was abolished by 5-Aza-dC and si-HGF (Fig. 4B and 4C). Therefore, high expression of HGF activated by HGF promoter methylation induced EMT, cell migration and invasion in NSCLC.

### DNA methylation of the HGF promoter in primary NSCLC allows prognosis evaluation in NSCLC

**Figure 5:**
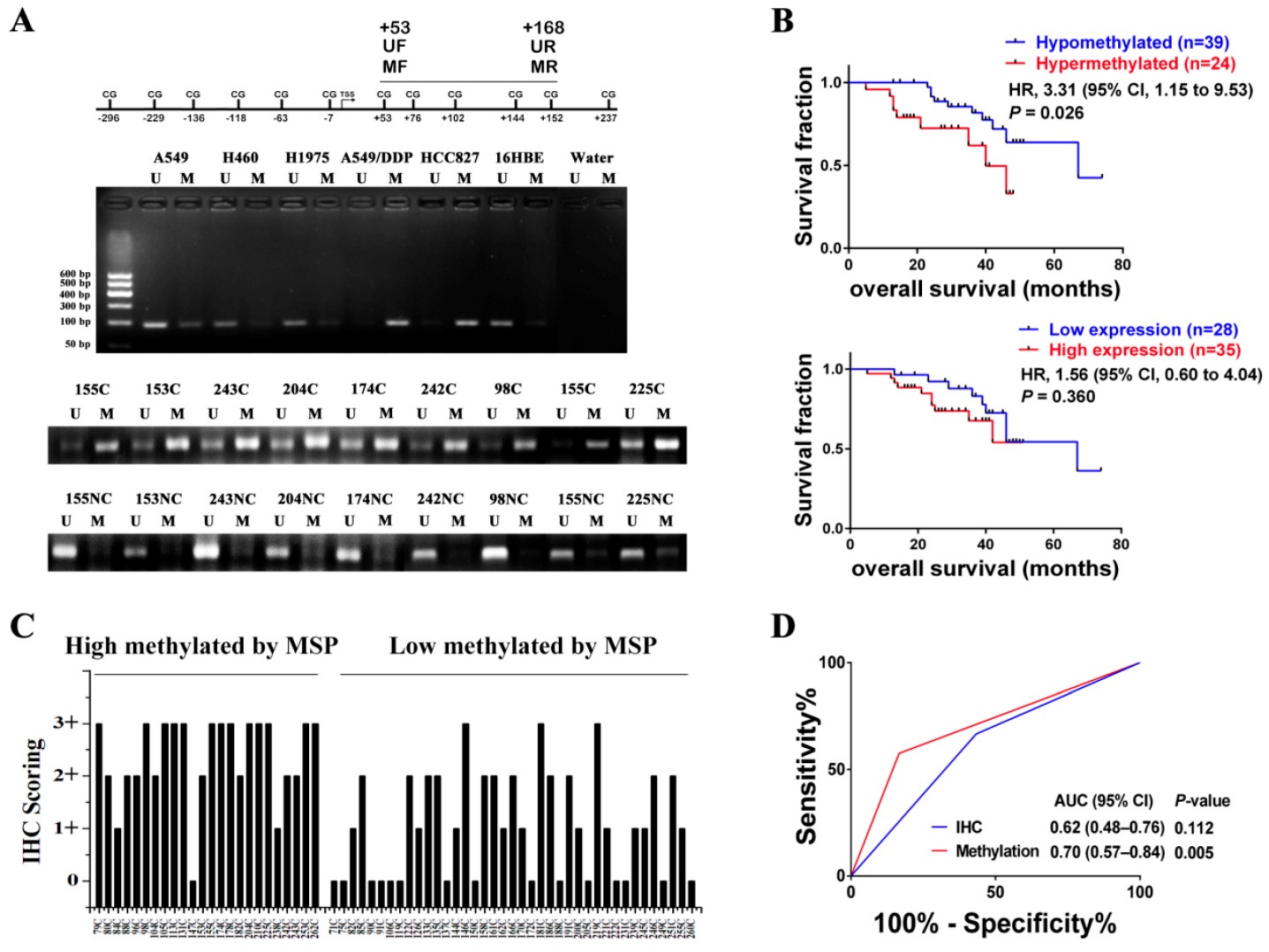
HGF promoter methylation levels were associated with poor prognosis of NSCLC. **(A)** Representative methylation-specific PCR (MSP) data for the HGF promoter region using the primer pairs represented in the schematic. M, methylated allele; U, unmethylated allele; N, normal lung samples; C, primary NSCLC samples. **(B)** Immunohistochemical (IHC) scoring of HGF expression in primary NSCLC samples identified as high methylation and low methylation by MSP. **(C)** Kaplan-Meier analysis of overall survival in NSCLC patients with primary tumors assessed for methylation levels of the HGF promoter and IHC scoring of HGF. The *P* values correspond to hazard ratios (HR). **(D)** ROC curves measuring the sensitivity and specificity of HGF promoter methylation and HGF protein in NSCLC to discriminate metastasis (n=34) from non-metastasis causes (n=30). MSP and IHC scores as categorical variables were used for the analysis, where HGF promoter methylation or HGF protein levels were dichotomized and the categories represented by 0 or 1 as follows: 0 (low risk) = low methylation (MI < 0.5) or low expression (IHC, 0 or 1+); 1 (high risk) = high methylation (MI ≥ 0.5) or high expression (IHC, 2+ or 3+). AUC and *P* values as specified.

In order to investigate whether HGF promoter methylation was associated with NSCLC, we measured the methylation status of the HGF promoter in 63 NSCLC samples using MSP analysis. The primers of MSP and UMSP were designed by MethPrimer 30, and only the region of +53 to +168 (including 4 CpGs: +53, +76, +102, +144) is suitable for detection because the HGF promoter contains less CpGs. The methylation of +53 CpG was also differential by Chip assay and pyrosequencing assay, and the methylation of +76 CpG was also differential by pyrosequencing assay (Fig. 1A). Here, the MSP results of 5 NSCLC epithelial cells (A549, A549/DDP, HCC827, H460 and H1975) and 1 normal lung epithelial cells (16HBE) were also detected, and the MSP results were consistent with the pyrosequencing results (Fig. 5A). For the NSCLC samples, the MSP results showed that 24 of 63 (38.1%) samples produced a MSP product with a methylation index (MI) score > 0.5 (Fig. 5A). Moreover, the RT-PCR assay which was used to detect HGF expression levels in the same 63 NSCLC samples showed significant positive correlations when HGF promoter methylation status (MI scores) was plotted against HGF expression levels (2-tailed Spearman's correlation, R = 0.529, *P* < 0.0001), suggesting that a highly methylated HGF promoter can activate the expression of HGF (Fig. S2). The 63 NSCLC samples were then divided into two groups depending on the MI scores (MSP results) of the HGF promoter, high methylation (MI ≥ 0.5) and low methylation (MI < 0.5). The association between HGF promoter methylation and the clinicopathologic features of NSCLC patients are listed in Table 1. Patients with high methylation were more likely to have stages III and IV (51.6% vs. 25.0%, *P*<0.05) and metastasis (57.5% vs. 16.7%, *P*<0.05) than patients with low methylation. Kaplan-Meier curves demonstrated that HGF promoter methylation was prognostic over the follow-up period, and the difference in mean overall survival (OS, 35.9 vs. 58.5 months) was statistically significant (*P*<0.05) (Fig. 5B). These findings suggested that HGF promoter methylation may be associated with an adverse prognosis of NSCLC by epigenetically activating HGF expression.

We also measured the protein expression of HGF (0, 1+, 2+, 3+) in the same 63 NSCLC samples by IHC (Fig. S3). As shown in Fig. 5C, in 21 of 24 (87.5%) high methylation samples, HGF protein levels were high. In contrast, in 14 of 39 (35.9%) low methylation samples, HGF protein levels were low. As mentioned above, it was also shown that the NSCLC samples with high methylation of the HGF promoter demonstrated correspondingly high expression levels of HGF. However, although patients with high HGF expression were more likely to have metastasis (48.0% vs. 16.7%) and a short mean OS (40.1 vs. 55.9) than patients with low HGF expression, the clinical correlation was not statistically significant (*P*>0.05) (Fig. 5B and Table S3). Moreover, we performed receiver operating characteristic curve (ROC) analysis of HGF promoter methylation to predict NSCLC metastasis and calculated the area under the curve (AUC) of the ROC to assess the sensitivity and specificity of the prediction: the AUC values were 0.70 (*P*<0.05) and higher than IHC of HGF (0.62, *P*>0.05) (Fig. 5D). Therefore, compared with IHC detection of HGF, detection of DNA methylation of the HGF promoter was significantly associated with tumor progression and shortened survival time in NSCLC patients.

## Discussion

As an inducible pleiotropic paracrine growth factor, HGF is generally induced and secreted by fibroblasts and acts via the MET receptor located mainly on epithelial cells, fulfilling important functions regarding cell proliferation, survival and motility, and is involved in wound healing and regeneration, angiogenesis, and the regulation of organogenesis in a variety of tissues 31, 32. Recently, HGF has been implicated in a wide range of malignancies including NSCLC, and aberrant high expression of HGF is related to a poor prognosis 33-35. Yang et al found that high expression of HGF can reduce the sensitivity to gefitinib in lung adenocarcinoma cells through MET and downstream PI3K and MAPK pathways 36. Yoneyama et al found that HGF was involved in the regulation of nicotine-induced cell migration by activating PI3K/Akt signaling pathway 37. Moreover, HGF produced by stromal fibroblasts also promote NSCLC cell survival, metastasis and tumor progression 38-40. As a biomarker, high levels of serum/plasma HGF may predict a poor prognosis in patients with NSCLC 41-44. As a therapeutic target, HGF inhibitor in combination with gefitinib or erlotinib has been used in clinical research of NSCLC 45, 46. In this study, a novel differential methylation region around the HGF promoter was identified in NSCLC, and positive correlations were observed between high methylation of the HGF promoter and high expression of HGF. Moreover, we also found that aberrant activation of HGF induced by high methylation of the HGF promoter was also involved in stimulating EMT and resulted in the migration and invasion of NSCLC epithelial cells. At present, there is growing evidence to suggest that DNA methylation has a close relationship with the occurrence and development of tumors and can be widely used as a tumor biomarker 47. In clinical applications, it was also observed that high methylation of the HGF promoter was associated with tumor progression and metastasis in NSCLC, indicating that HGF promoter methylation status can potentially act as a biomarker in NSCLC. Therefore, although an independent cohort study and more detailed clinical analysis, such as clinical setting of TKI-resistant disease, were needed to be conducted in future studies, epigenetic detection of the HGF promoter may be a new biomarker or may be combined with other biomarkers for NSCLC diagnosis and therapy.

In general, HGF is expressed by fibroblasts but not by epithelial cells in normal lung tissue 18. Although aberrant high expression of HGF has been found in NSCLC epithelial cells, the molecular mechanisms of aberrant HGF expression in NSCLC epithelial cells are still unclear. In the present study, DNA methylation analysis of the HGF gene showed that HGF expression can be activated in NSCLC epithelial cells with high methylation of the HGF promoter compared with NSCLC epithelial cells with medium or low methylation of the HGF promoter. This indicated that methylation status of the HGF promoter was involved in the regulation of HGF expression in NSCLC epithelial cells. It has been reported that DNA methylation can influence the expression of HGF, and some studies have found that 5-Aza-dC can strongly reduce the expression of HGF *in vivo* 48-50. Furthermore, it has been reported that HGF treatment upregulated the expression of DNA methyltransferase 1 (DNMT1) which induced DNA hypermethylation and the expression of HGF can also be stimulated by itself though its receptor MET 51, 52. Here, we found that 5-Aza-dC treatment can reduce the HGF expression and inhibit EMT, although 5-Aza-dC induced DNA demethylation may result in an indirect impact on the HGF expression and EMT. Therefore, combined with our findings that high methylation of the HGF promoter activated HGF expression, it is suggested that self-activation of HGF through its receptor MET may be associated with HGF‑induced DNA hypermethylation which may also enhance methylation of the HGF promoter.

It is well known that the regulation of gene expression through epigenetic mechanisms is a complex phenomenon 53. By preventing transcriptional activators binding or recruiting methyl CpG-binding proteins (MBP) and thereby abolishing transcription initiation, DNA methylation is often associated with transcriptional block, silencing of transposable elements and heterochromatin formation 54. Recently, specific DNA methylation was also found to be involved in the activation of gene expression. CpG methylation of the CRE sequence (TGACGTCA) enhances DNA binding of the C/EBPα transcription factor, a protein critical for activation of differentiation in various cell types 55. CTCF, which binds DNA in a methylation-sensitive manner, is able to block enhancer function, and BCL6 expression can be maintained during lymphomagenesis in part through DNA methylation which prevents CTCF-mediated silencing 56. There are two possible mechanisms for the promotion of gene expression by DNA methylation. On the one hand, the methylated gene promoter could active gene transcription by recruiting transcription factors which specifically bind to the methylated DNA promoter 55. On the other hand, the methylated gene promoter also has the potential to positively regulate gene transcription, albeit in an indirect manner, by preventing transcriptional repressors binding and thereby increasing transcription 56. In the present study, under certain induction conditions, the methylated HGF promoter increased the binding of RNA Pol II compared with the unmethylated HGF promoter, suggesting that DNA methylation of the HGF promoter facilitates the binding and function of RNA Pol II on the HGF promoter in NSCLC epithelial cells.

In the ENCODE project 57, it was found that CpGs around the HGF promoter were unmethylated both in normal lung fibroblasts (IMR90 and AG04450 cells) and normal lung epithelial cells (HPAEpiC, SAEC, and NHBE cells). Therefore, some transcriptional repressors may be present in epithelial cells to prevent RNA Pol II binding. Presumably, as DNA methylation levels of the HGF promoter increased during NSCLC progression, the methylated HGF promoter can facilitate the binding of RNA Pol II by inhibiting the binding of repressors on the HGF promoter, and then NSCLC epithelial cells have the potential to induce the expression of HGF. Moreover, cisplatin resistant A549/DDP cells were induced from A549 cells, which indicated that increased methylation of the HGF promoter in A549/DDP cells can be induced by cisplatin treatment. High expression of HGF has also been reported to be associated with cisplatin resistance in NSCLC 58. Therefore, we suggest that, under various conditions such as chemotherapeutic drugs and the cell microenvironment, NSCLC epithelial cells with HGF promoter methylation can be induced to activate HGF which promoted drug resistance and metastasis of NSCLC epithelial cells and led to poor prognosis in NSCLC patients. Such novel molecular mechanisms which sustain NSCLC have the potential of shedding light on NSCLC and provide a molecular basis for the disease processes. However, additional studies are required to examine the specific formation of repressive chromatin structures on the HGF promoter in lung epithelial cells.

## Conclusion

In summary, it was observed that DNA methylation of the HGF promoter was associated with aberrant activation of HGF expression in NSCLC epithelial cells and played a significant role in the EMT of NSCLC epithelial cells which resulted in progression and metastasis of NSCLC. Furthermore, understanding the molecular mechanisms of HGF promoter methylation may also provide insights and methods for the treatment of NSCLC.

## Supplementary Material

Supplementary figures and tables.

## Figures and Tables

**Table 1:** Correlation between DNA methylation of HGF promoter and the clinicopathological features of NSCLC patients. (*P<0.05)

Clinicopathological factors		DNA methylation level^1^ of HGF promoter		χ² value	*P* value
		High methylation	Low methylation		
**Histological type**	Adenocarcinoma	13	19	0.177	0.674
	Squamouscarcinoma	11	20		
**Gender**	Male	19	30	0.042	0.837
	Female	5	9		
**Age (years)**	<60	12	19	0.010	0.921
	≥60	12	20		
**Smoking**	Nonsmoker	15	18	1.593	0.207
	Smoker	9	21		
**TNM Clinical stage**	I	2	8	11.482*	0.009*
	II	6	16		
	III	7	13		
	IV	9	2		
**Metastasis**	0	5	25	11.157*	0.001*
	1	19	14		
**Relapse**	0	7	21	3.671	0.055
	1	17	18		

1. DNA methylation level of HGF promoter was used the MI scores as categorical variables, where HGF promoter methylation level was dichotomised and its categories represented as follows: cases with MI ≥ 0.5 were designated as “high methylation”, whereas cases with MI < 0.5 were designated as “low methylation”.
