# Supplementary material for: Epigenetic activation of hepatocyte growth factor is associated with epithelial-mesenchymal transition and clinical outcome in non-small cell lung cancer
Source: J Cancer. 2019 Aug 28;10(21):5070–81. doi: 10.7150/jca.30034 (PMC6775597; doi:10.7150/jca.30034)
Supplement: Supplementary file 1 — Supplementary figures and tables. [file jcav10p5070s1.pdf]

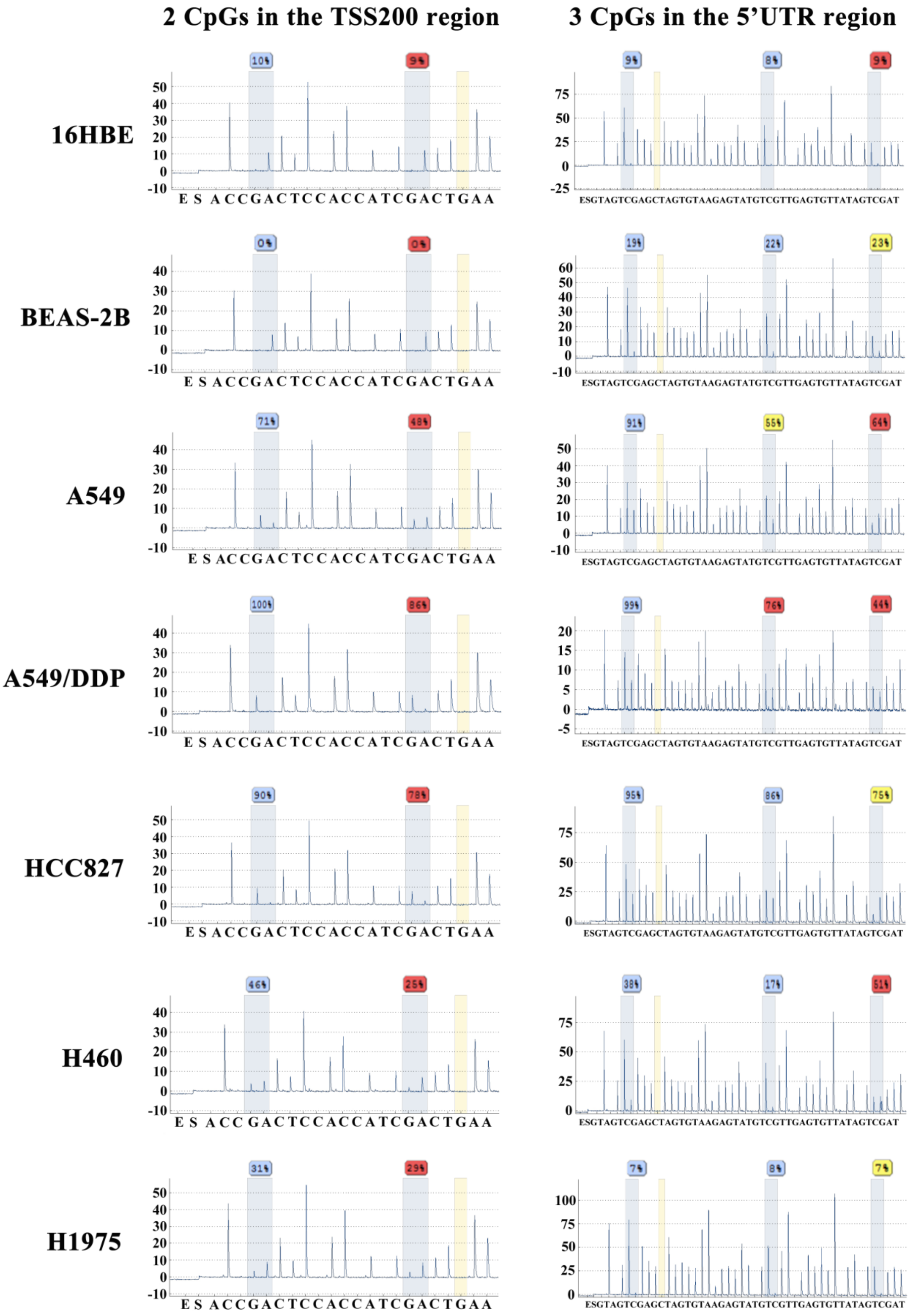

Figure S1. Pyrosequencing analysis results of HGF promoter.

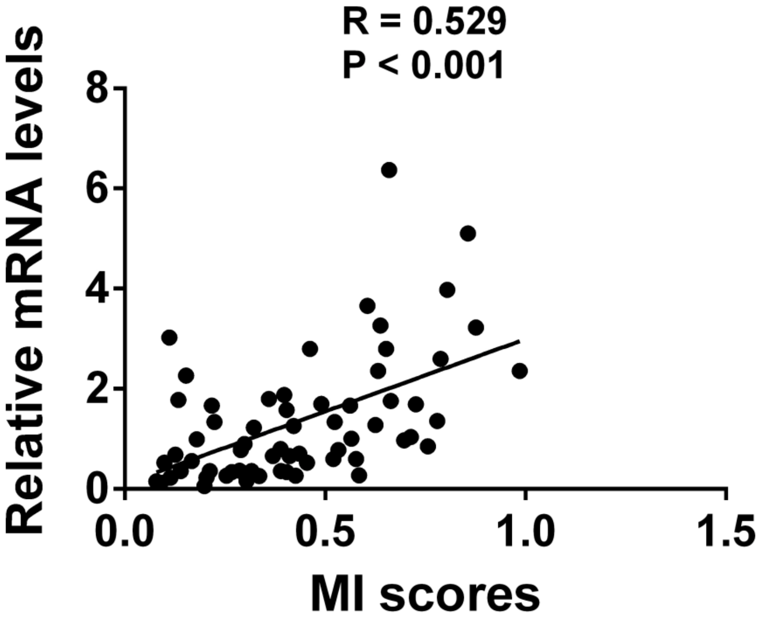

Figure S2. Correlation between the HGF promoter methylation status and the HGF expression levels in 63 NSCLC patients, expressed using the linear regression (solid line).

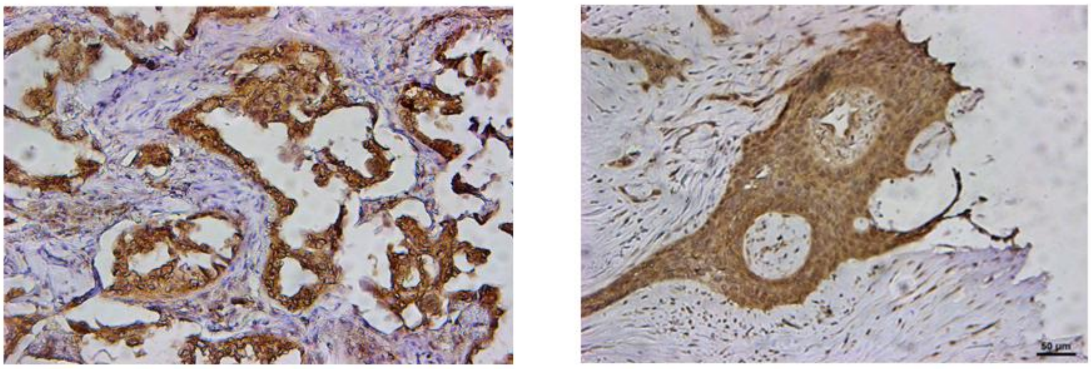

Adenocarcinoma Squamouscarcinoma

Figure S3. Representative HGF immunohistochemistry (IHC) results of primary NSCLC. Primary adenocarcinoma and squamouscarcinoma tissues with strong HGF protein staining in tumor cells.

Table S1. Different methylation of HGF between carcinoma tissues (C) and paracarcinoma tissues (NC) of 5 NSCLC patients (S1-S5).

	**Gene region**	**Fold change of *β*-values (C/NC)**				
		**S1**[1]	**S2**	**S3**	**S4**	**S5**
1	TSS1500	1.07	0.93	0.77	1.04	0.99
2	TSS1500	**1.58**	0.95	**0.58**	0.92	1.19
3	TSS1500	**2.02**	1.22	0.90	0.94	0.86
4	TSS200	**2.00**	**2.05**	0.96	0.98	**2.32**
5	TSS200	1.43	**1.98**	**1.56**	1.17	**1.59**
6	5'UTR	**1.51**	**1.72**	1.06	0.99	**2.07**
7	5'UTR	1.34	1.22	0.76	1.04	1.41
8	Gene body	1.06	0.91	0.80	1.05	0.97
9	Gene body	0.97	0.98	0.78	0.95	0.91
10	Gene body	1.01	0.91	0.74	1.04	0.93

1. Clinicopathological features of 5 patients: S1, adenocarcinoma, female, 57, nonsmoker, T2aN2M0 (stage III); S2, adenocarcinoma, male, 59, smoker, T3N2M0 (stage III); S3, adenocarcinoma, male, 40, smoker, T2aN0M0 (stage I); S4, adenocarcinoma, female, 69, nonsmoker, T1N0M0 (stage I); S5, adenocarcinoma, female, 72, nonsmoker, T2aN2M0 (stage III).

Table S2. Pyrosequencing analysis of the methylation status of HGF promoter in 5 NSCLC epithelial cells (A549, A549/DDP, HCC827, H460 and H1975 cells) and 2 normal lung epithelial cells (16HBE and BEAS-2B cells).

	Cells	Methylation percentages (%)				
		Position -136[1]	Position -118	Position 53	Position 76	Position 102
1	16HBE	9	10	9	8	9
2	BEAS-2B	0	0	23	22	19
3	A549	48	71	64	55	91
4	A549/DDP	86	100	44	76	99
5	HCC827	78	90	75	86	95
6	H460	25	46	51	17	38
7	H1975	29	31	7	8	7

1. Pyrosequencing assay was designed for detecting HGF promoter at 2 CpGs in the TSS200 region (position -136 and -118) and 3 CpGs in the 5’UTR region (position 53, 76 and 102) (chromosome 7, GRCh37/hg19). The transcription start site (TSS) was set as position 1.

Table S3. Correlation between the expression of HGF and the clinicopathological features of NSCLC patients.

Clinicopathological factors		Expression level[1] of HGF protein		χ^2 value	*P* value
		High	Low		
Histological type	Adenocarcinoma	17	15	0.156	0.692
	Squamouscarcinoma	18	13		
Gender	Male	27	22	0.018	0.893
	Female	8	6		
Age (years)	<60	16	15	0.383	0.536
	≥60	19	13		
Smoking	Nonsmoker	20	13	0.719	0.397
	Smoker	15	15		
TNM Clinical stage	I	5	5	4.112	0.250
	II	12	10		
	III	9	11		
	IV	9	2		
Metastasis	0	13	17	3.471	0.062
	1	22	11		
Relapse	0	14	16	1.837	0.175
	1	21	12		

1. Expression level of HGF was used the IHC scores as categorical variables, where HGF protein level was dichotomised and its categories represented as follows: cases with scores of 2+ or 3+ were designated as “high”, whereas cases with scores of 0 or 1+ were designated as “low”.

Table S4. Overall patient characteristics

Clinicopathological factors	
**Total:**	
N	68
**Gender, n (%):**	
Male	53 (77.9%)
Female	15 (22.1%)
**Age, years (range):**	
Mean	59.2 (29-81)
**Histological type, n (%)**	
Adenocarcinoma	37 (54.4%)
Squamouscarcinoma	31 (45.6%)
**TNM Clinical stage, n (%)**	
I	12 (17.6%)
II	25 (36.8%)
III	20 (29.4%)
IV	11 (16.2%)
